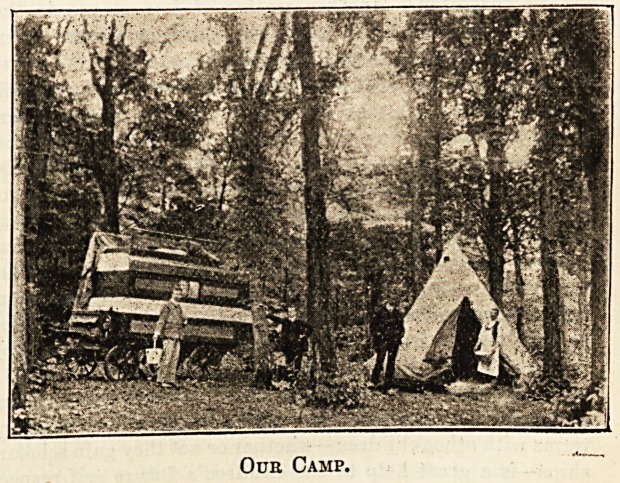# A Week in a Caravan

**Published:** 1908-07-25

**Authors:** 


### The Practitioner's Relaxations and Hobbies.

### A WEEK IN A CARAVAN.

It was with a sigh of relief that I, in the late autumn, at length tore myself away from my country practice and fled, looking fearfully back, lest some patient might even at the eleventh hour grasp at my coat-tails and drag me back to drudgery.

A caravan holiday, a sort of "inland voyage," was the programme this year, in company with three kindred spirits. I had some little difficulty in selecting suitable companions. This must be very carefully done in a caravan tour, as you are to be very

Our Caravan.

much thrown together, and also because, as you are expected to take a hand in the household work, it is important to select people whom you know will be willing to take the biggest share in it.

Taking train to Edinburgh, we cabbed to our caravan. We were very pleased with the equipage. It had originally been a furniture-van, but, being intended for nobler uses, had been transformed until I am sure it did not know itself. It was a work of art. At first I thought we had lighted on an ice-cream shop on wheels. No one could live in a house like that and be dull.

Two small windows had been let into the side, and these were ornamented with beautiful little curtains hung on yellow ribbon. The door at the back had windows that opened like those of a railway carriage. The firm's name and address were pasted over with broad strips of brightly-coloured paper the entire length of the van. The top was guarded by a rail a foot and a half high. Inside, at the front end, were two broad shelves which could be used as beds at night. On each side were seats that could also be made into beds, and there were also two hammocks. A table occupied the centre.

It was a dull enough afternoon when we set out. The rain, which had fallen heavily all the forenoon, ceased, but the rolling clouds, obscuring the face of the sun, gave little promise of fair weather. An hour's journey brought us into the open country. We camped that night at the little village of Eddleston, near Peebles. We went to bed, but not to sleep; we had not yet got used to the decorated furniture-van. The wind howled and the rain fell in torrents. I thought we were going to have a repetition of the Flood, and wondered if the old ark was watertight.

Then I became restless and turned over; this shook the van. Some one else turned over, and the van shook again. Then some one began to snore; by the time we got him awake again the rain ceased.

When morning broke I found we were in a little wood in the midst of the village. By the side of the wood rippled the Eddleston water. Raindrops clung to the foliage, sparkling and scintillating like diamonds in the morning light. The sun, gleaming through the trees, shed patches of light on the brown carpet of the wood.

Breakfast was a complicated affair. It took three cooks, but they did not spoil the broth. The grate, being an open one, was inclined to be smoky sometimes. Occasionally a chop burst jubilantly into flame; it was none the worse of that, just overdone a little. Being in a hurry, we arranged the washing of the dishes to take place as we went along. Two of our number walked behind the caravan at the open door. On the floor was placed a tub full of water. The dishes were handed down to our aristocratic member, who washed them and handed them to the tall gentleman of our party. Jehu knew nothing of this pleasant little arrangement, and being in a hurry, started our fiery steed at a trot.

Faster and faster went the horse, faster and faster had to go in consequence the unlucky pair. A sudden

Our Camp.

jolt of the van and out flew basin of soapy water, dishes, knives, forks, and spoons on top of them, bringing the operations to a sudden stop.

After passing through Peebles and Innerleithen, we took the mountain road to Ettrickvale. This road crosses the Tweed at Innerleithen and enters a sylvan district. It begins among trees, it leads to the bare heathery wilds; it begins in the valley, it winds over the shoulder of the mountain. Up, up we go, mile after mile, and ever as we lift our eyes the hills rise before us, until, reaching a height of

1,153 feet above sea-level, we gain the summit and dismiss the trace horse.

That silver thread, meandering along the floor of the valley, hidden at times as it winds round some shady corner, reappearing to glitter with renewed brilliancy as the sun's rays dip into its waters once more—that is the Yarrow, sung by the poets.

That part of the Yarrow to which we now descended flows along the floor of a bare and open valley. We turned to the right on reaching the Gordon Arms Inn, and were shortly within sight of St. Mary's Loch. The famous Tibbie Shiel's Inn is placed just between St. Mary's Loch and the Loch of the Lowes. "Christopher North," the author of the well-known *Noctes Ambrosianæ*, came here many a time to fish for salmon trout, and to have a night with the "Ettrick Shepherd."

The bleat of the sheep comes with strangely human note, floating across the valley as if the place were peopled with the ghosts of generations that have gone. One might almost imagine it to be the voice of a large and mighty conventicle of the hunted and martyred Covenanters, many of whom sojourned in these wilds; or perhaps the fairies and brownies were busy that evening. At any rate, we came away feeling we had been near the Unseen and Eternal.

## Figures and Tables

**Figure f1:**
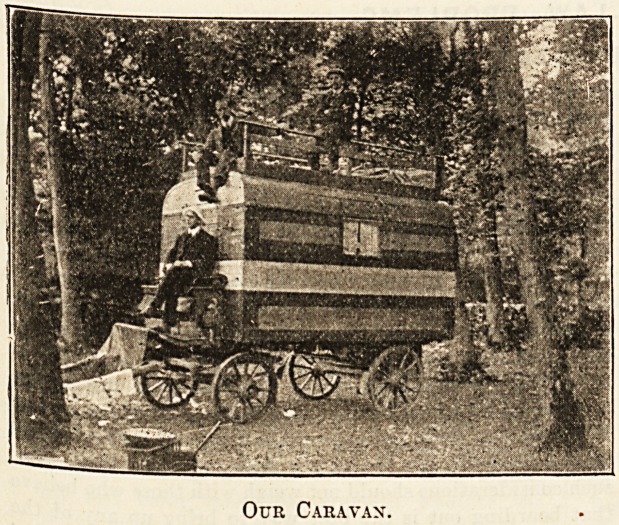


**Figure f2:**